# Effects of dietary inclusion of blooming *Ulva* sp. on milk production, methane emitting potential, and physiological parameters in lactating Holstein cows

**DOI:** 10.5713/ab.25.0060

**Published:** 2025-06-04

**Authors:** Kiyeon Park, Yoorae Kim, Eungseok Kim, Jongchul Lee, Weonjong Yoon, Kyewon Kang, Seongwon Seo, Honggu Lee

**Affiliations:** 1 Department of Animal Science and Technology, Sanghuh College of Life Science, Konkuk University, Seoul, Korea; 2 IANS Co., Ltd., Cheonan, Korea; 3 Jeju Biodiversity Research Institute, Jeju Technopark, Jeju, Korea; 4 Division of Animal and Dairy Sciences, Chungnam National University, Daejeon, Korea

**Keywords:** Algae, CH_4_, Cortisol, Dairy, Green Tide, Seaweed

## Abstract

**Objective:**

*Ulva* sp., a blooming macroalgae causing the green tide in Korea, has been suggested as a feed ingredient for ruminant livestock. The objective of this study was to investigate the effects of 3% dry matter inclusion of *Ulva* sp. in a total mixed ration (TMR) on milk production, methane emitting potential, and physiological parameters in lactating Holstein cows.

**Methods:**

A total of 36 cows were allocated into two groups considering milk production, parity, days in milk, and methane production concentration from respiration and eructation, and fed the TMR with or without 3% dry matter inclusion of *Ulva* sp. for 4 weeks. Methane emitting potential was measured using a laser methane detector in week 4, and feed, milk, and blood were collected every 2 weeks.

**Results:**

*Ulva* sp. inclusion did not affect methane production concentration from respiration and eructation, but tended to decrease the methane intensity concentration from respiration (p = 0.06) and eructation (p = 0.06). In addition, it increased the milk fat yield, energy-corrected milk, and net energy for lactation in week 2, but this did not persist to week 4, indicating the interaction between treatment and week (p<0.05). Likewise, it increased milk urea nitrogen, blood urea nitrogen, and white blood cell counts in week 2 but not in week 4 (p<0.05). Cortisol concentration in hair tended to decrease with *Ulva* sp. inclusion (p<0.10), whereas the serum total antioxidant capacity and uric acid were not affected.

**Conclusion:**

It was demonstrated that *Ulva* sp. can be utilized as a feed ingredient for lactating cows without any adverse effects on milk production, complete blood cell counts, or blood metabolites. The potential methane-reducing property of *Ulva* sp. should be further investigated in future studies.

## INTRODUCTION

In the livestock industry, rumen CH_4_ mitigation is one of the most intensively studied areas, as the CH_4_ emission from ruminant enteric fermentation accounts for 39.1% of greenhouse gas emissions from the livestock supply chain and 5.7% of total anthropogenic greenhouse gas emissions [1]. Among the various approaches to mitigate rumen methane emission, seaweeds have shown profound CH_4_-mitigating effects, especially *Asparagopsis* sp. [2–4]. In this regard, the CH_4_ mitigation properties of various seaweed species from Korea have been extensively investigated [5–7].

A massive bloom of *Ulva* sp., also called the green tide, occurred in China and Korea and incurred considerable disposal costs and environmental damage [8,9]. In this regard, large amounts of *Ulva* sp. were collected, but there was no effective strategy for the utilization of the collected biomass. As a solution, our research team suggested utilizing *Ulva* sp. as a ruminant feed ingredient [7]. *Ulva* sp. and *U. ohnoi* harvested from tropical coast of Australia reduced rumen methane emissions when incubated *in vitro* [4]. It was assumed that ulvan, the sulfated polysaccharides of *Ulva* sp. induced the methane mitigating effects [10–12]. However, the effects of *Ulva* sp. cultivated in laboratory on the methane reduction was observed in *in vitro* batch culture, but not in continuous culture [3,13]. These inconsistent results could be due to the different sampling condition of the seaweed. Chemical composition of seaweeds can vary by the geographical and seasonal variations [14–16]. Therefore, study investigating the effects of *Ulva* sp. collected in temperate coast of Northeast Asia in ruminant diet is required. In our previous study, *Ulva* sp. bloomed in Korea were included in up to 4% dry matter (DM) to make an isoenergetic and isoproteic diet and then incubated in in situ and *in vitro* [7]. As a result, the *Ulva* sp. decreased the rumen methane production and proportion and did not affect total volatile fatty acids production up to 3% DM inclusion. However, further effects of *Ulva* sp. on dairy productivity and physiology, especially antioxidant and immunomodulating properties, remain undetermined.

The objective of the present study was to demonstrate the effects of the dietary inclusion of *Ulva* sp. on (1) milk production, (2) methane emitting potential, and (3) physiological parameters in lactating Holstein cows. Based on our previous study, a 3% DM inclusion rate was chosen [7]. It was hypothesized that (1) *Ulva* sp. inclusion does not impair milk production in lactating cows, (2) *Ulva* sp. may reduce rumen methane emitting potential, and (3) *Ulva* sp. inclusion may have antioxidant and immunomodulating effects in lactating cows.

## MATERIALS AND METHODS

### Animals, diet, and experimental design

All animal procedures were approved by the Institution of Animal Care and Use Committee at Konkuk University (Approval no. KU21141). A total of 36 lactating Holstein cows (milk yield: 33.37±5.82 kg d^−1^; parity: 2.03±1.17; days in milk: 198.64±62.09 d; methane production concentration from respiration, MPCR: 33.94±8.21 ppm m^−1^; methane production concentration from eructation, MPCE: 133.74±66.75 ppm m^−1^) were allocated into two groups considering parity, days in milk, milk production, MPCE, and MPCR. The cows were fed with or without 3% DM inclusion of *Ulva* sp. in the total mixed ration (TMR) for 4 weeks. In addition to the TMR, all cows were fed 1.84 kg DM of concentrate and 0.57 kg DM of rye silage.

All feeds were sampled every 2 weeks and analyzed in triplicate. Ether extract (Method 920.39) and crude ash (Ash; Method 942.05) were analyzed as described by AOAC [17]. The neutral detergent fiber (NDF) and acid detergent fiber were analyzed according to the procedure of Van Soest et al [18] using a heat-stable amylase with residual ash. The acid detergent lignin content was analyzed as described by Jung et al [19]. Nitrogen contents, including crude protein (CP), neutral detergent insoluble CP (NDICP), and acid detergent insoluble CP (ADICP), were analyzed using the procedure of AOAC [17]; Method 954.01). The CP, NDICP, and ADICP contents of *Ulva* sp. were estimated using a specific nitrogen-to-protein conversion factor of 5.13 [20]. Nitrogen in NDF (NDF_N_) was calculated by subtracting NDICP from NDF. The net energy for lactation (NE_L_) was calculated as described in NRC [21]. The chemical compositions of the diets are shown in Table 1.

### *Ulva* sp. collection

*Ulva* sp. blooming in Korea were manually collected in Seongsan-eup, Seogwipo-si, Jeju-do, Republic of Korea, from September 2020 to August 2021. The collected *Ulva* sp. was roughly dried at 40°C–45°C for 48–96 h and then thoroughly air-dried at 55°C for 72 h. The dried *Ulva* sp. was pulverized and then mixed into the TMR.

### Sampling and lab analysis procedure

Enteric CH_4_ emissions were measured on the last week of the trial using a laser methane detector (LMD, mini-G[50A], Tokyo Gas Engineering), as described by Kang et al [22]. In brief, the exhaled CH_4_ concentration (ppm m^−1^) of each cow was measured 4 times a day (−2, −1, +1, and +2 h after morning feeding) for 6 min. The data were processed using an Automatic Multi-scale Peak Detection algorithm to separate the peaks into respiration and eructation. Then, MPCR and MPCE were analyzed and reported. The average of each of the 4 measurements was presumed to be the most representative daily CH_4_ production from each cow. The MPCR and MPCE are expressed as ppm m^−1^, and the methane intensity concentration from respiration (MICR) and the methane intensity concentration from eructation (MICE) are expressed as ppm m^−1^ per kg milk yield per day.

Milk samples were collected during 2 consecutive milking events, pooled in equal volume with 2-bromo-2-nitropropane-1,3 diol (Broad Spectrum Bicrotabs 2; Advanced Instruments), and analyzed every 2 weeks using MilkoScan FT1 (FossAlle 1 DK – 3400).

Blood samples were collected from jugular veins every 2 weeks before feeding. Complete blood cell counts (CBCs) were analyzed using VetScan HM2 (Abaxis). Serum metabolites, including glutamic oxaloacetic transaminase (GOT), glutamic pyruvic transaminase (GPT), blood urea nitrogen (BUN), creatine, albumin, total protein, triglycerides, and uric acid, and plasma metabolites, including total cholesterol, low-density lipoprotein (LDL), high-density lipoprotein (HDL), lactate dehydrogenase (LDH), and glucose, were analyzed using a DRI CHEM 7000i biochemistry analyzer (FUJIFILM). Serum total antioxidant capacity (TAC) was analyzed using an assay kit (MBS2540515; MyBioSource) according to the manufacturer’s protocol using spectrophotometry (Synergy2; biotek).

The hair grown during the experiment was prepared according to the procedure described by Nejad et al [23]. The hair was ground using a bead beater (tacoPrep Bead Beater, ATPB-01; GeneReach Biotechnology) and analyzed using a cortisol ELISA kit (1-3002; Salimetrics) according to the manufacturer’s protocol.

### Statistical analysis

The data sampled repeatedly, including milk production, CBCs, and blood metabolites, were analyzed using the MIXED procedure of SAS software (SAS Institute) with the sources of *Ulva* sp. inclusion, week, and their interaction as a fixed factor, and animal as a random factor. The covariance structure with the lowest AIC value was chosen among the CS, CSH, AR(1), ARH(1), TOEPH, and ANTE(1) statements. The other data, including methane production and intensity, hair cortisol, and TAC, were analyzed with the source of *Ulva* sp. inclusion as a fixed factor, and animal within a group as a random factor. All values are reported as LSMEANS. A significant difference was accepted when p<0.05, and the tendency was accepted when 0.05≤p<0.10.

## RESULTS

### Milk production

**Figure 1:**
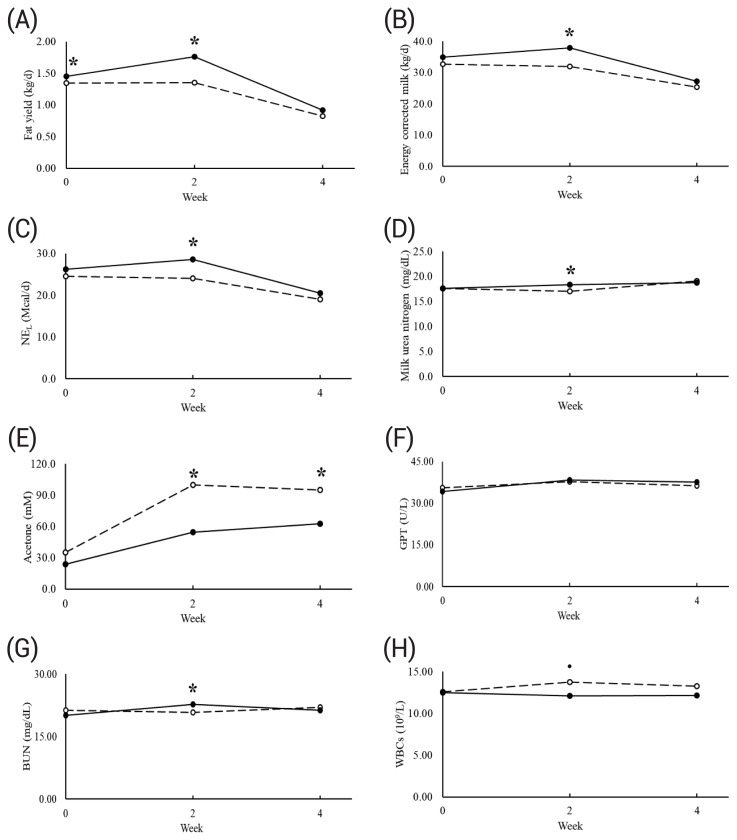
Effects of dietary 3% DM inclusion of *Ulva* sp. on milk fat yield (A), energy-corrected milk (B), net energy for lactation, NE_L_ (C), milk urea nitrogen (D), milk acetone (E), glutamic pyruvic transaminase (F), blood urea nitrogen (G), and white blood cell counts (H) in lactating cows. Black represents the group with *Ulva* sp. inclusion, and white dot represents the control group. * significant difference (p<0.05); ·, tendency (0.05≤p<0.10). NE_L_, net energy for lactation; GPT, glutamic pyruvic transaminase; BUN, blood urea nitrogen; WBC, white blood cell count; DM, dry matter.

The effects of 3 % DM inclusion of *Ulva* sp. on milk productivity are shown in Table 2. The milk yield, proportion of milk fat, protein, lactose, lactose yield, and somatic cell counts were not affected by *Ulva* sp. An interaction between treatment and week was detected for the milk fat yield (p = 0.04), energy-corrected milk (ECM; p<0.05), NE_L_ (p<0.05), milk urea nitrogen (MUN; p = 0.01), and milk acetone (p = 0.01). In detail, *Ulva* sp. inclusion increased the milk fat yield in week 2, but there were no significant differences in week 4 (Figure 1). Likewise, ECM, NE_L_, and MUN increased with *Ulva* sp. inclusion in week 2, but there were no significant differences in week 4. *Ulva* sp. inclusion decreased milk acetone in weeks 2 and 4. However, *Ulva* sp. inclusion decreased milk beta-hydroxybutyrate (BHB) regardless of the week (p<0.05).

### CH**_4_** emission, hair cortisol, and total antioxidant capacity

The effects of 3 % DM inclusion of *Ulva* sp. on methane emission are shown in Table 3. *Ulva* sp. inclusion did not affect MPCR or MPCE. However, it tended to decrease MICR (16.9%; p = 0.06) and MICE (14.2%; p = 0.06). Hair cortisol was also decreased numerically by *Ulva* sp. (17.6%; p = 0.10; Table 3). There was no effect of *Ulva* sp. inclusion on TAC.

### Blood metabolites

The effects of 3 % DM inclusion of *Ulva* sp. on blood metabolites are shown in Table 4. An interaction between treatment and week was detected for GPT, but there were no significant differences between the control and treatment groups throughout the whole experiment (p = 0.01; Figure 1). *Ulva* sp. inclusion increased BUN in week 2, but there was no difference in week 4 (p<0.01). An interaction tendency was detected in glucose (p = 0.07) and HDL (p = 0.09). However, there were no significant differences between the control and treatment groups in each week. The main effects of *Ulva* sp. inclusion or interactions were not detected for GOT, creatine, albumin, total protein, triglycerides, uric acid, total cholesterol, LDH, or LDL.

### Complete blood cell counts

The effects of 3 % DM inclusion of *Ulva* sp. on blood metabolites are shown in Table 5. An interaction between *Ulva* sp. and week was detected for white blood cell counts (WBCs; p = 0.04). In detail, *Ulva* sp. inclusion tended to decrease WBCs in week 2 (p = 0.08), but not in week 0 or 4 (Figure 1). The proportions of lymphocytes, monocytes, and granulocytes were not affected by the main effect of *Ulva* sp. inclusion or interactions. However, *Ulva* sp. inclusion tended to decrease the number of granulocytes (13.0 %; p = 0.10) throughout the 4 weeks. In addition, an interaction tendency was detected for the mean corpuscular volume (p = 0.07), but there were no significant differences between the control and treatment groups throughout the 4 weeks. Likewise, red blood cell counts, hematocrit, hemoglobin, mean corpuscular hemoglobin, mean corpuscular hemoglobin concentration, platelet, mean platelet volume, mean platelet volume, and platelet crit were not affected by the main effect of *Ulva* sp. inclusion or interactions.

## DISCUSSION

The previous study by our research team investigated the nutritional value of *Ulva* sp. *in vitro* and *in situ* [7]. Total volatile fatty acids production was not changed by *Ulva* sp. inclusion up to 3% dietary DM with linear decreasing CH_4_ production. Also, DM effective rumen degradability was similar to that of alfalfa hay (61.3 vs. 62.2 %/h). In our preliminary trial, we demonstrated there was no palatability issue. Therefore, it was presumed that 3% DM inclusion of *Ulva* sp. did not impair intake, rumen fermentation, and digestibility. However, the lack of intake, rumen microbiome, and digestibility data are the limitations of this study. Therefore, the results of this study should be interpreted with caution, especially regarding the methane-reducing property.

### Effects of *Ulva* sp. dietary inclusion on methane emissions

In our previous study, *Ulva* sp. bloomed in Korea linearly decreased the production and proportion of methane after 48 h of incubation [7]. Likewise, *Ulva* sp. harvested from the tropical ocean [4] or cultivated in the lab [3,13] also decreased rumen *in vitro* CH_4_ emission. In the current *in vivo* study, MICR and MICE tended to decrease as a result of *Ulva* sp. inclusion, which is in line with our second hypothesis that *Ulva* sp. inclusion may reduce rumen methane emitting potential. To the best of our knowledge, this is the first study to report the methane-mitigating potential of blooming *Ulva* sp. in lactating cows. However, methane production, which was reported as MPCR and MPCE, was not affected by the treatment. This could be because LMD data can be influenced by various environmental factors, such as wind speed and direction, relative humidity, and pressure [24]. Meanwhile, the persistency of the anti-methanogenic effects of *Ulva* sp. is still unclear because this study was conducted for 4 weeks, and methane emission was not repeatedly measured. Ulvan, a water-soluble sulfated polysaccharide that represents 8%–29% of the dry weight of *Ulva* sp., has been suggested to be a major bioactive compound of *Ulva* sp. [10,11]. With this value, the estimated ulvan contents fed to cows was 0.24%–0.87% of dietary DM. However, rumen microbes can adapt to some feed additives. Guan et al [25] reported that 33 mg kg^−1^ of monensin administration decreased 30% of methane emissions in beef cattle, which was restored within 2 months. Wu et al [26] reported that rumen microbes started to adapt to citrus essential oil in terms of methane emissions after 3 weeks of administration. Bacteroidetes phylum has a wide range of carbohydrate-active enzymes, which can degrade algal-derived sulfated polysaccharides, such as fucoidan [12,27]. Therefore, the possibility of rumen microbial adaptation to ulvan remains, which could be related to the temporary effect of *Ulva* sp. on the milk fat yield described later. A long-term study is required to examine the microbial adaptation to *Ulva* sp. with repeated measurements of CH_4_ emissions and rumen microbial analysis.

### Effects of *Ulva* sp. dietary inclusion on dairy productivity

Milk yield, milk protein, and lactose yield and proportion were not affected by *Ulva* sp., which is consistent with our first hypothesis. The milk fat yield, ECM, and NE_L_ increased as a result of *Ulva* sp. inclusion in week 2 but did not persist to week 4. It is difficult to ascertain whether the increased milk fat yield, NE_L_, and ECM were due to the additional energy consumption with *Ulva* sp., as the energy content of *Ulva* sp. is comparatively lower than that of the TMR due to its high ash amounts. Therefore, we suggest that the increased milk fat yield, ECM, and NE_L_ in week 2 were partially due to the saved energy by *Ulva* sp. such as methane mitigation. Up to 12% of feed gross energy could be partially redirected to milk production via CH_4_ mitigation [28]. Kinley et al [2] reported that 0.10% OM of *Asparagopsis taxiformis* reduced 38% of CH_4_ production with an increase in average weight gain. This is partially supported by the reduced milk acetone in this study. Milk acetone is an indicator of hyperketonemia in dairy cows [29]. Although the acetone levels in both groups were far below the hyperketonemia level (1.4 mM), reduced milk acetone showed the potential of *Ulva* sp. in ketosis prevention by revising the energy balance in lactating cows. However, our previous study reported that the proportion of acetate linearly decreased with the *Ulva* sp. inclusion rate and that the proportion of propionate linearly increased [7], making it difficult to explain the increased milk fat yield and the maintained milk and lactose yield. Likewise, Maia et al [3] reported that a 25 DM% addition of cultivated *Ulva* sp. increased the acetate proportion with CH_4_ mitigation in the rumen in an *in vitro* batch culture system. Furthermore, MICR and MICE decreased up to 15% by *Ulva* sp. in this study. However, MPCR and MPCE were not changed. In addition, ECM increased around 3.3 kg/d by *Ulva* sp., which is hard to explain only with the CH_4_ reduction. If the increased milk fat yield was not via the reduced methanogenesis, then it could be due to its effects on the rumen lipid metabolism and de novo milk fat synthesis in the mammary gland. Considering the low-fat content of *Ulva* sp. (0.21% DM), it is difficult to presume that the fatty acid profile of *Ulva* sp. directly affected the milk fat yield. The antimicrobial property of ulvan [10, 11] in *Ulva* sp. could possibly impact rumen biohydrogenation microbes such as *Butyrivibrio fibrisolvens* and *Butyrivibrio proteoclasticus*, and decrease alternative biohydrogenation pathway represented by *trans*-10, *cis*-12 C18:2 and *trans*-10 C18:1. Huang et al [30] reported that phlorotannin extract from *Sargassum* sp. inhibited rumen biohydrogenation and elevated carbohydrate-utilizing bacteria in the rumen. Likewise, algal bioactive compounds such as ulvan could impact rumen lipid metabolism, resulting in the milk fat yield. Therefore, the impact of ulvan on rumen lipid metabolism is worthwhile to be investigate in future studies. If the effect of *Ulva* sp. on milk fat yield was via CH_4_ reduction or altering rumen lipid metabolism, it disappeared in week 4. It indicates that its effects on the rumen microbiome were temporary probably due to the microbial adaptation. Longer *in vivo* trials might be required focusing on the rumen microbiome in the future.

Milk urea nitrogen increased as a result of *Ulva* sp. inclusion in week 2. This could be due to the high nitrogen uptake in the treatment group, which resulted in increased BUN. Our previous study reported that *Ulva* sp. has a high RUP (54.64% of CP) compared to alfalfa hay (28.57% of CP), which might be due to the high NDICP content [7]. We suggested that the high NDICP content might have originated from the high amounts of glycoproteins in the cell wall of *Ulva* sp. [31]. The increased MUN and BUN imply that the high RUP of *Ulva* sp. can be digested and absorbed in lactating cows. Gaillard et al [32] also reported that the total amino acids of *Ulva* sp. collected from northern Norway have 27.4%, 51.2%, and 78.6% ruminal, small intestinal, and total tract degradability, and they concluded that *Ulva* sp. can be considered as relevant protein sources for ruminants. However, they did not result in high albumin or total protein in the serum and milk in the present study. This is consistent with the study by Rjiba-Ktita et al [33], who reported that the 20–40 DM% inclusion of *Ulva* sp. collected from Tunisia did not alter the average daily weight gain, total tract CP digestibility, or the nitrogen balance in lambs. To understand the discrepancy between the increased MUN and BUN, maintained albumin, and the total protein in serum and milk, additional parameters, including urinary and fecal nitrogen excretion, should be measured in future studies.

### Effects of *Ulva* sp. dietary inclusion on blood metabolites, hair cortisol, and complete blood cell counts

Despite the significant interaction between the treatment and week affecting the serum GPT level, there were no significant differences between the control and treatment group throughout the experiment. Likewise, serum GOT and creatine were not affected by *Ulva* sp. inclusion, indicating that *Ulva* sp. did not impact liver and kidney function [34,35]. Additionally, the plasma LDH level was not affected by *Ulva* sp., indicating that *Ulva* sp. did not affect overall tissue damage and inflammation in lactating cows [36]. Meanwhile, *Ulva* sp. tended to decrease WBCs in week 2 by reducing the number of granulocytes, which might be related to the immunomodulating effect of ulvan [11]. Kim et al [37] found that 80% ethanol extraction from *Ulva* sp. increased nitric oxide and TNF-α in RAW 264.7 macrophages, which also could be resulted from the immunomodulating property of ulvan. However, it is difficult to assert that the possible immunomodulating property of *Ulva* sp. affected the dairy productivity in this study, as all cows maintained a healthy status without any clinical symptoms. More straightforward clinical studies should be conducted in the future to demonstrate the immunomodulating function of *Ulva* sp. in lactating cows.

An antioxidant capacity of *Ulva* sp. has been suggested [38]. We hypothesized that the antioxidant capacity of *Ulva* sp. may contribute to decreasing the stress level in lactating cows. *Ulva* sp. inclusion showed a tendency to decrease hair cortisol levels by 17.6%, which shows the potential stress alleviation effects of *Ulva* sp. in lactating cows. This is partially consistent with the study by Ellamie et al [39], who reported that the heat-stress-alleviating effects of *Sargassum latifolium* in sheep alleviated anemia and dyslipidemia and improved the antioxidant defense system and inflammatory response. However, TAC and uric acid were not affected by *Ulva* sp. in this study, which is inconsistent with our third hypothesis. Therefore, the detailed mechanism of the stress-relieving effect of *Ulva* sp. in lactating cows is still unclear, and it should be reinvestigated in the future.

## CONCLUSION

Taken together, dietary 3% DM inclusion of blooming *Ulva* sp. in Korea tended to decrease the methane intensity concentration in lactating cows, which was consistent with our hypothesis. It also increased milk productivity by increasing the milk fat yield, NE_L_, and ECM, but it did not persist for 4 weeks. Likewise, it increased MUN and BUN, but this also did not persist for 4 weeks. Rumen microbial adaptation to *Ulva* sp. needs to be investigated in a long-term *in vivo* trial. In addition, *Ulva* sp. inclusion seemed to alleviate stress in lactating cows by decreasing hair cortisol levels, whereas TAC and uric acid were not affected. Although the detailed mechanism of the effect of *Ulva* sp. on milk production and hair cortisol is still not clear, this study demonstrated that *Ulva* sp. can be utilized as a feed ingredient for lactating cows without any adverse effects on milk production, CBC, or blood metabolites with potential CH_4_ reducing property. Therefore, we strongly suggest utilizing *Ulva* sp. from the green tide as a dairy cow feed ingredient for the sustainable dairy industry.

## Figures and Tables

**Table 1:** Chemical compositions of diet total mixed ration for control and treatment groups (3 DM% inclusion of *Ulva* sp.), concentrate, rye silage, and *Ulva* sp.

Parameters	TMR	Concentrate	Rye silage	*Ulva* sp.
Analyzed values (%, dry matter basis)				
CP^1)^	16.5±0.10	20.36±1.60	7.79±1.96	15.26±0.08
NDF	43.77±0.27	27.34±0.20	69.92±0.96	36.21±0.48
NDICP	6.08±0.77	13.54±0.39	1.25±0.18	10.45±0.31
ADF	22.25±1.37	10.45±0.26	44.13±0.86	15.22±0.30
ADICP	3.59±0.25	5.76±0.42	1.08±0.04	3.73±0.41
ADL	4.12±0.13	1.72±0.34	6.58±2.50	5.68±0.38
EE	4.44±0.32	4.29±0.18	3.51±0.51	0.21±0.01
Ash	7.57±0.06	7.65±0.31	8.32±0.84	30.48±0.09
Calculated values				
NDF_N_	37.69	13.80	68.67	25.76
NE_L_	1.56	1.77	1.15	0.85

Analyzed values are presented as mean±standard deviation.

1) CP for *Ulva* sp. was estimated with a specific nitrogen-to-protein conversion factor of 5.13 [20].

DM, dry matter; TMR, total mixed ration; CP, crude protein; NDF, neutral detergent fiber; NDICP, neutral detergent insoluble crude protein; ADF, acid detergent fiber; ADICP, acid detergent insoluble crude protein; ADL, acid detergent insoluble lignin; EE, ether extract; NDFN, NDF – NDICP (%, dry matter basis); NE_L_, net energy for lactation (Mcal kg DM^−1^).

**Table 2:** Effects of dietary 3% DM inclusion of *Ulva* sp. on milk yield and composition in lactating cows

Parameters	CON	TRT	SEM	p-value		

				Trt	Wk	Trt×Wk
Milk yield (kg d^−1^)	31.2	32.7	0.54	0.41	<0.01	0.18
Fat (%)	3.87	4.25	0.142	0.18	<0.01	0.17
Protein (%)	3.23	3.23	0.019	0.95	<0.01	0.11
Lactose (%)	4.72	4.76	0.013	0.38	0.63	0.56
Fat yield (kg d^−1^)	1.17	1.38	0.045	0.01	<0.01	0.04
Protein yield (kg d^−1^)	1.00	1.05	0.016	0.36	<0.01	0.55
Lactose yield (kg d^−1^)	1.47	1.55	0.026	0.34	<0.01	0.74
ECM yield (kg d^−1^)^1)^	30.0	33.3	0.66	0.03	<0.01	0.05
NE_L_ yield (Mcal kg^−1^)^2)^	22.6	25.1	0.50	0.03	<0.01	0.05
Somatic cells counts (10^3^ mL^−1^)	240	308	81.1	0.73	0.07	0.58
MUN (mg dL^−1^)	17.9	18.2	0.19	0.57	<0.01	0.01
Acetone (μM)	76.7	47.0	0.01	0.02	<0.01	0.01
BHB (μM)	85.4	78.7	0.00	0.05	<0.01	0.11

1) Milk yield (kg/d)×({383×milk fat [%]+242×milk protein [%]+165.4×milk lactose [%]+20.7}/3,140) [40].

2) 9.29×milk fat yield (kg/d)+5.85×milk protein yield (kg/d)+3.95×milk lactose yield (kg/d) [41].

DM, dry matter; SEM, standard error of the mean; Trt, main effect of the treatment; Wk, main effect of week; Trt×Wk, interaction between treatment and week; ECM, energy-corrected milk; NE_L_, net energy for lactation; MUN, milk urea nitrogen; BHB, β-hydroxybutyrate.

**Table 3:** Effects of dietary 3% DM inclusion of *Ulva* sp. on methane production, intensity, hair cortisol, and total antioxidant capacity in lactating cows

Parameters	CON	TRT	SEM	p-value
MPCR (ppm m^−1^)	37.4	36.0	1.89	0.71
MPCE (ppm m^−1^)	155	150	5.8	0.67
MICR (ppm m^−1^ kg milk yield^−1^)	1.27	1.06	0.059	0.06
MICE (ppm m^−1^ kg milk yield^−1^)	5.24	4.50	0.198	0.06
Hair cortisol (pg mg^−1^)	5.77	4.76	0.309	0.10
Total antioxidant capacity (U mL^−1^)	5.05	4.52	0.211	0.22

DM, dry matter; SEM, standard error of the mean; MPCR, methane production concentration from respiration; MPCE, methane production concentration from eructation; MICR, methane intensity concentration from respiration; MICE, methane intensity concentration from eructation.

**Table 4:** Effects of dietary 3% DM inclusion of *Ulva* sp. on blood metabolites, including glutamic oxaloacetic transaminase, glutamic pyruvic transaminase, blood urea nitrogen, creatine, albumin, total protein, triglycerides, uric acid, total cholesterol, low-density lipoprotein, high-density lipoprotein, lactate dehydrogenase, and glucose in lactating cows

Parameters	CON	TRT	SEM	p-value		

				Trt	Wk	Trt×Wk
GOT (U L^−1^)	72.8	73.5	1.08	0.85	0.02	0.19
GPT (U L^−1^)	36. 6	36.7	0.46	0.91	<0.01	0.01
BUN (mg dL^−1^)	21.4	21.3	0.24	0.97	<0.01	<0.01
Creatine (μg dL^−1^)	668	648	9.2	0.44	<0.01	0.75
Albumin (g dL^−1^)	3.73	3.72	0.022	0.83	0.34	0.69
Total protein (g dL^−1^)	7.70	7.65	0.040	0.67	0.03	0.46
Triglycerides (mg dL^−1^)	4.46	4.63	0.268	0.81	0.02	0.29
Uric acid (U L^−1^)	1.23	1.18	0.017	0.26	<0.01	0.11
Glucose (mg dL^−1^)	42.1	43.3	0.75	0.52	<0.01	0.07
TCHO (mg dL^−1^)	323	329	5.6	0.72	<0.01	0.54
LDH (U L^−1^)	958	979	19.0	0.67	<0.01	0.25
HDL (mg dL^−1^)	216	227	5.4	0.56	<0.01	0.09
LDL (mg dL^−1^)	107	103	2.6	0.38	0.13	0.70

DM, dry matter; SEM, standard error of the mean; GOT, glutamic-oxaloacetic transaminase; GPT, glutamic pyruvic transaminase; BUN, blood urea nitrogen; TCHO, total cholesterol; LDH, lactate dehydrogenase; HDL, high-density lipoprotein; LDL, low-density lipoprotein.

**Table 5:** Effects of dietary 3% DM inclusion of *Ulva* sp. on complete blood cell counts in lactating cows

Parameters	CON	TRT	SEM	p-value		

				Trt	Wk	Trt×Wk
WBCs (10^9^ L^−1^)	13.2	12.3	0.32	0.37	0.47	0.04
Lymphocytes (10^9^ L^−1^)	8.12	7.76	0.305	0.72	0.31	0.23
Lymphocytes (%)	60. 8	62.2	1.22	0.70	0.22	0.35
Monocytes (10^9^ L^−1^)	0.594	0.634	0.0557	0.70	<0.01	0.43
Monocytes (%)	4.69	5.48	0.433	0.38	<0.01	0.55
Granulocytes (10^9^ L^−1^)	4.44	3.87	0.139	0.10	0.12	0.24
Granulocytes (%)	35.1	32.3	1.01	0.36	0.05	0.25
RBCs (10^12^ L^−1^)	7.11	7.38	0.076	0.24	<0.01	0.93
Hemoglobin (g dL^−1^)	11.1	11.2	0.11	0.67	<0.01	0.61
Hematocrit (%)	31.7	32.1	0.28	0.71	<0.01	0.69
MCV (fL)	44.7	43.6	0.30	0.29	0.67	0.07
RDWc (%)	19.6	19.9	0.09	0.24	<0.01	0.89
MCH (pg)	15.6	15.3	0.10	0.44	0.01	0.19
MCHC (g dL^−1^)	34.8	35.1	0.10	0.28	0.01	0.82
Platelet (109 L^−1^)	381	420	29.4	0.52	0.03	0.66
MPV (fL)	7.20	7.21	0.054	0.95	0.13	0.65
PCT (%)	0.274	0.292	0.0151	0.56	0.04	0.65
PDWc (%)	32.4	33.0	0.21	0.33	0.25	0.18

DM, dry matter; SEM, standard error of the mean; WBCs, white blood cells; RBCs, red blood cells; MCV, mean corpuscular volume; RDWc, red cell distribution width; MCH, mean corpuscular hemoglobin; MCHC, mean corpuscular hemoglobin concentration; MPV, mean platelet volume; PCT, platelet crit; PDWc, platelet distribution width.
